# Discrete time domain modeling and design of current mode controlled flyback LED driver

**DOI:** 10.1038/s41598-023-33162-7

**Published:** 2023-04-18

**Authors:** Marn-Go Kim

**Affiliations:** School of Electrical Engineering, Pukyong National University, Busan, Republic of Korea

**Keywords:** Electrical and electronic engineering, Characterization and analytical techniques

## Abstract

In this paper, modeling for a current mode controlled (CMC) flyback LED driver with a stabilizing ramp is performed in a step-by-step procedure. Discrete time state equations for the system are derived and linearized with respect to a steady-state operating point. At this operating point, the switching control law, the condition that determines the duty ratio, is also linearized. In the next step, a closed-loop system model is derived by combining the two models of the flyback driver and the switching control law. The root locus analysis in the z-plane is used to investigate the characteristics of the combined linearized system and obtain design guidelines for feedback loops. The feasibility of the proposed design is confirmed through the experimental results for the CMC flyback LED driver.

## Introduction

In recent years, light emitting diode (LED) lighting becomes increasingly popular owing to the advantages of its high luminous efficacy, environmental friendliness, long lifespan, and fast response^1^. The brightness of an LED is mainly controlled by the forward current of the LED supplied by a current converter. Due to the manufacturing tolerance of forward voltage and internal resistance of individual LEDs, accurately controlling the forward current is an important issue. Therefore, it is necessary to control the current to get the brightness of the LEDs precisely^2–4^. A lot of work has been done recently for the applications of power LEDs, including rectification with a high-power factor^5–11^ and current balancing between LED strings^12–14^.

Several small-signal model approaches have been attempted to characterize current-mode controlled converters. A low-frequency average approach was proposed in^15^. The modulator model is derived from the perturbation of an equation for the average inductor current in steady-state. This model has gained wide acceptance owing to its simplicity. However, one general limitation of the averaging model is its inability to predict high-frequency small-signal dynamics near half of the switching frequency. Discrete-time models^16^ can accurately predict the response at high frequencies, but provide little design insight due to their complex formulas based on numerical techniques. So far, when modeling the behavior of the modulator, it has been assumed that the operation of the control signal ${v}_{c}$ in the output feedback loop is much slower than the switching operation. This approach is suitable for voltage regulating converters because a low-pass filter is added in the output.

Current control is required to accurately maintain the brightness of the LEDs. The control signal ${v}_{c}$ in the output feedback loop of the current controlled converter is fast. Therefore, it cannot be regarded as a constant over a switching period. This is because a low-pass filter for the feedback output current is not necessarily required. In the current regulating converter, the instantaneous control signal ${v}_{c}$ in the output feedback loop must be used to model the behavior of the modulator. In order to improve the characteristics of the LED driver, an optimal design of the circuits in the feedback loops is required. However, very little research has been done in this area^17–21^.

**Figure 1:**
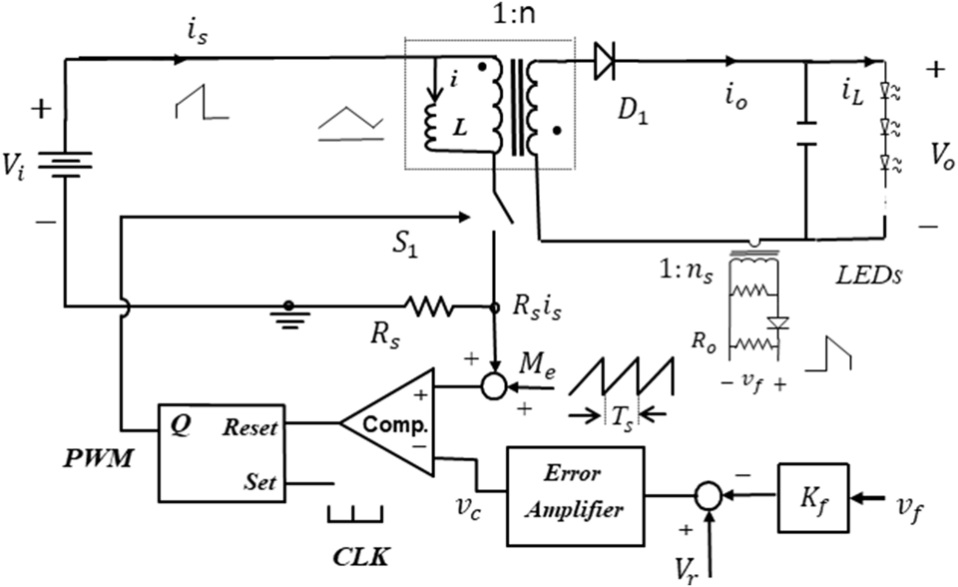
Constant-frequency CMC flyback LED driver with stabilization ramp.

In this paper, modeling and analysis of the CMC flyback LED driver shown in Fig. 1 is performed using a systematic modeling technique^22–25^. The root locus analysis in the z-plane is utilized to derive the stability boundary as a function of D. Design guidelines for feedback loops such as stabilization ramp slope and integrator gain are presented in a step-by-step process. In particular, this paper reveals for the first time that the proportional gain of the proportional-integral (PI) error amplifier in the output current feedback loop is not related to the dynamic characteristics of the CMC flyback LED driver. This is because, when the switch is on, the output current of the flyback converter is 0, so the slope change of the control signal by the output current does not occur. The validity of the proposed analysis and design is confirmed through transient response experiments.

## Modeling of CMC flyback LED driver

The following assumptions are utilized to perform the discrete time domain model:

- The flyback converter is operating in continuous conduction mode (CCM),
- All switches are ideal,
- The loading effect on the power stage by the feedback loop is negligible,
- The input and output voltages are DC.

### Discrete-time state equations

The switch-on state is initiated when a clock signal is applied to the Set input of the PWM modulator. The switched-on state ends when the sum of the stabilization ramp ${M}_{e}$ and the inductor current sense signal ${R}_{s}{i}_{s}$ reaches the control signal ${v}_{c}$.

The duty ratio ${d}_{k}$ is determined when the two comparator inputs are equal as follows

1 $${R}_{s}\left({i}_{k}+{m}_{1}{T}_{s}{d}_{k}\right)+{M}_{e}{T}_{s}{d}_{k}={v}_{c}{|}_{t={t}_{k}+{T}_{s}{d}_{k}}$$

where ${i}_{k}$ is the primary inductor current at the beginning of the k-th cycle, ${M}_{e}$ is the external ramp slope added for stabilization, and ${m}_{1}$ is the positive slope of inductor current when the power switch is ON.

**Figure 2:**
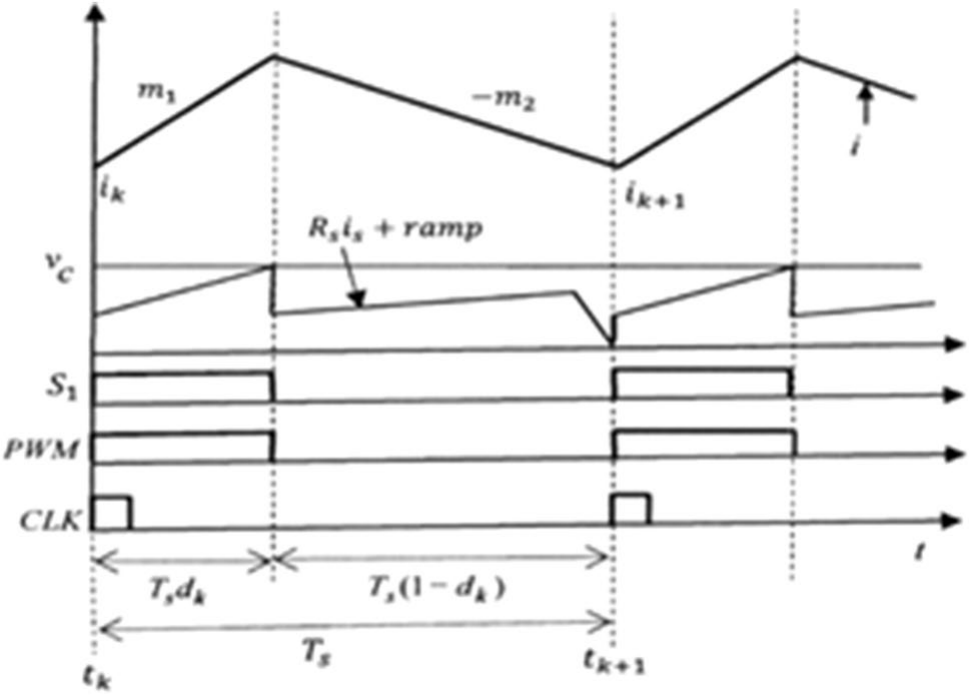
Key typical waveforms of Fig. 1.

In Fig. 2, the inductor current can be expressed in the discrete time domain by the equation

2 $${i}_{k+1}={i}_{k}+{m}_{1}{T}_{s}{d}_{k}-{m}_{2}{T}_{s}(1-{d}_{k})$$

where ${i}_{k+1}$ is the primary inductor current at the end of the k-th cycle, and ${m}_{2}$ is the negative slope of inductor current when the power switch is OFF.

**Figure 3:**
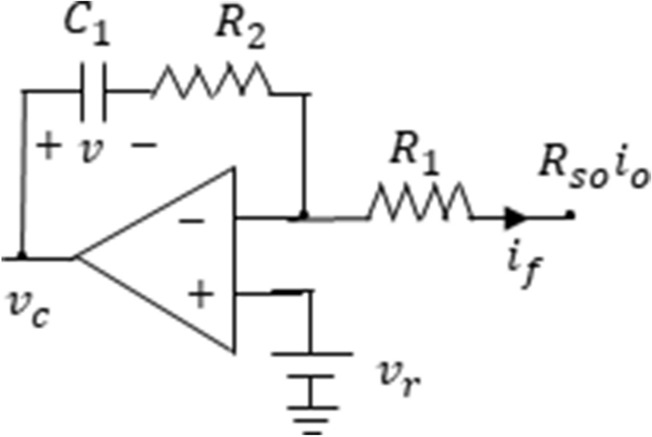
PI error amplifier in the output feedback loop.

As the error amplifier circuit of the output current feedback loop, a PI compensator is used as shown in Fig. 3. The capacitor voltage of the error amplifier can be written in the discrete time domain by the equation

3 $$ \begin{aligned} &v_{k + 1}  = v_{k} + \frac{1}{{C_{1} }}\mathop \int \limits_{{t_{k} }}^{{t_{k + 1} }} \frac{{\left( {v_{r} - R_{so} i_{o} } \right)}}{{R_{1} }}dt \\&\quad = v_{k} + \frac{{R_{so} T_{s} }}{{R_{1} C_{1} }}\left( {\frac{{v_{r} }}{{R_{so} }} - i_{avg,k} } \right) \\ \end{aligned} $$

where ${R}_{so}=\frac{{R}_{o}{K}_{f}}{{n}_{s}} , {i}_{avg,k}=\frac{1}{{T}_{s}}{\int }_{{t}_{k}}^{{t}_{k+1}}{i}_{o}dt$, ${n}_{s}$ is the turns ratio of output current sense transformer, ${K}_{f}$ is the output current feedback gain and ${R}_{o}$ is the output current sense resistor.${i}_{avg,k}$, which is the average output current of k-th cycle, can be calculated as

4 $${i}_{avg,k}=\frac{1}{n}\{{i}_{k}+{m}_{1}{T}_{s}{d}_{k}-{m}_{2}\frac{{T}_{s}\left(1-{d}_{k}\right)}{2}\}\cdot (1-{d}_{k})$$

where n is the turns ratio of the flyback transformer.

Since ${i}_{o}$ is 0 for ${t}_{k}\le t<{t}_{k}+{T}_{s}{d}_{k}$, the control signal $v_{c}$ at the moment of switching can be represented as

5 $$ \begin{aligned} & v_{c} |_{{t = t_{k} + T_{s} d_{k} }}  = v_{r} + \frac{{R_{2} }}{{R_{1} }}v_{r} + v_{k} + \frac{1}{{C_{1} }}\mathop \int \limits_{{t_{k} }}^{{t_{k} + T_{s} d_{k} }} \frac{{v_{r} }}{{R_{1} }}{\text{dt}} \\& = v_{r} + k_{p} \cdot v_{r} + v_{k} + k_{i} T_{s} d_{k} \cdot v_{r} \\ \end{aligned} $$

where the P gain ${k}_{p}$ is $\frac{{R}_{2}}{{R}_{1}}$ and the I gain ${k}_{i}$ is $\frac{1}{{R}_{1}{C}_{1}}$. When ${v}_{r}$ is constant, the slope of the control signal at the switching moment is not affected by ${k}_{p}$.

### Equilibrium state

Setting ${m}_{1}={M}_{1}$, ${m}_{2}={M}_{2}$,${d}_{k}=D$,${i}_{k+1}={i}_{k}=I$, ${v}_{k+1}={v}_{k}=V$, ${i}_{avg,k}={I}_{avg}$ ${\mathrm{i}}_{\mathrm{avg},\mathrm{k}}={\mathrm{I}}_{\mathrm{avg}}$ ${\mathrm{i}}_{\mathrm{avg},\mathrm{k}}={\mathrm{I}}_{\mathrm{avg}}$, and ${v}_{r}={V}_{r}$, the following steady-state conditions can be obtained from (2) and (3):

6a $${M}_{1}{T}_{s}D={M}_{2}{T}_{s}(1-D)$$

6b $${I}_{avg}={V}_{r}/{R}_{so}$$

where $$\mathrm{D}=\frac{{V}_{o}/n}{{V}_{i}+{V}_{o}/n}$$, $${M}_{1}={V}_{i}/L$$ and $${M}_{2}=\frac{{V}_{o} /n}{L}$$.

Using (4), the average output current ${I}_{avg}$ at equilibrium state can be written as

7 $${I}_{avg}=\frac{1}{n}\left[I+{M}_{1}{T}_{s}D-\frac{{M}_{2}{T}_{s}\left(1-D\right)}{2} \right]\left(1-D\right)=\frac{{V}_{r}}{{R}_{so}}$$

### Linearization

Equations (2) and (3) can be expressed concisely as

8a $${i}_{k+1}={f}_{1}[{i}_{k}{,v}_{k},{d}_{k},{v}_{r}]$$

8b $${v}_{k+1}={f}_{2}[{i}_{k}{,v}_{k},{d}_{k},{v}_{r}]$$

The switching control law can also be expressed from Eqs. (1) and (5) as

9 $${f}_{3}\left[{i}_{k}{,v}_{k},{d}_{k},{v}_{r}\right]=0$$

Linearizing Eq. (8a) with respect to the operating point gives

10 $$\updelta {i}_{k+1}=\frac{\partial {f}_{1}}{\partial {i}_{k}}{|}_{*}\cdot \delta {i}_{k}+\frac{\partial {f}_{1}}{\partial {v}_{k}}{|}_{*}\cdot \delta {v}_{k}+\frac{\partial {f}_{1}}{\partial {d}_{k}}{|}_{*}\cdot \delta {d}_{k}+\frac{\partial {f}_{1}}{\partial {v}_{r}}{|}_{*}\cdot \delta {v}_{r}$$

where

$$\frac{\partial {f}_{1}}{\partial {i}_{k}}{|}_{*}=1,$$ $$\frac{\partial {f}_{1}}{\partial {v}_{k}}{|}_{*}=0$$, $$\frac{\partial {f}_{1}}{\partial {d}_{k}}{|}_{*}=\left({M}_{1}+{M}_{2}\right){T}_{s}=\frac{{V}_{i}+{V}_{o}/n}{{f}_{s}L}$$,

$$\frac{\partial {f}_{1}}{\partial {v}_{r}}{|}_{*}=0$$.

Using steady-state conditions, linearizing Eq. (8b) with respect to the operating point also gives

11 $$\updelta {v}_{k+1}=\frac{\partial {f}_{2}}{\partial {i}_{k}}{|}_{*}\cdot \delta {i}_{k}+\frac{\partial {f}_{2}}{\partial {v}_{k}}{|}_{*}\cdot \delta {v}_{k}+\frac{\partial {f}_{2}}{\partial {d}_{k}}{|}_{*}\cdot \delta {d}_{k}+\frac{\partial {f}_{2}}{\partial {v}_{r}}{|}_{*}\cdot \delta {v}_{r}$$

where

$$\frac{\partial {f}_{2}}{\partial {i}_{k}}{|}_{*}=-{k}_{i}{T}_{s}{R}_{so}(1-D)/n,\frac{\partial {f}_{2}}{\partial {v}_{k}}{|}_{*}=1$$,

$$\frac{\partial {f}_{2}}{\partial {d}_{k}}{|}_{*}=-{k}_{i}{T}_{s}\cdot \frac{{R}_{so}}{n}[\left({M}_{1}{T}_{s}+\frac{{M}_{2}{T}_{s}}{2} \right)\cdot (1-D)-\frac{{V}_{r}}{{R}_{so}}\cdot \frac{n}{1-D}]$$,

$$\frac{\partial {f}_{2}}{\partial {v}_{r}}{|}_{*}={k}_{i}{T}_{s}$$.

Linearizing the switching control law in Eq. (9) can be derived as

12 $$ \frac{{\partial f_{3} }}{{\partial i_{k} }}|_{*} \cdot \delta i_{k} + \frac{{\partial f_{3} }}{{\partial v_{k} }}|_{*} \cdot \delta v_{k} + \frac{{\partial f_{3} }}{{\partial d_{k} }}|_{*} \cdot \delta d_{k} + \frac{{\partial f_{3} }}{{\partial v_{r} }}|_{*} \cdot \delta v_{r} = 0 $$

where

$$\frac{\partial {f}_{3}}{\partial {i}_{k}}{|}_{*}=1,$$ $$\frac{\partial {f}_{3}}{\partial {v}_{k}}{|}_{*}=-\frac{1}{{R}_{s}}$$,$$\frac{\partial {f}_{3}}{\partial {d}_{k}}{|}_{*}={M}_{1}{T}_{s}+\frac{{M}_{e}{T}_{s}}{{R}_{s}}-{K}_{i}{T}_{s}\cdot \frac{{V}_{r}}{{R}_{s}}$$

$$\frac{\partial {f}_{3}}{\partial {v}_{r}}{|}_{*}=-(1+{k}_{p}+{k}_{i}{T}_{s}D)/{R}_{s}$$.

### Combination of state equations and control law

Equation (12) can be used to derive the following form for the linearized control law:

13 $$\updelta {d}_{k}=-{K}_{1}\cdot \delta {i}_{k}-{K}_{2}\cdot \delta {v}_{k}-{K}_{3}\cdot \delta {v}_{r}$$

where

$${K}_{1}=\frac{1}{{M}_{1}{T}_{s}+\frac{{M}_{e}{T}_{s}}{{R}_{s}}-{k}_{i}{T}_{s}\cdot \frac{{V}_{r}}{{R}_{s}}}, {K}_{2}=-\frac{{K}_{1}}{{R}_{s}} ,$$ and

$${K}_{3}=-\frac{1+{k}_{p}+{k}_{i}{T}_{s}D}{{R}_{s}}\cdot {K}_{1}$$

Substituting (13) into (10) and (11) yields the following closed-loop system:

14 $$\updelta {X}_{k+1}=A\cdot \delta {X}_{k}+B\cdot \delta {v}_{r}$$

where ${\updelta }X_{k + 1} = [ \delta i_{k + 1} \delta v_{k + 1} ]^{T}$, ${\updelta }X_{k} = [ \delta i_{k} \delta v_{k} ]^{T}$. $${\text{A}} = \left[ {\begin{array}{*{20}c} {a_{11} } & {a_{12} } \\ {a_{21} } & {a_{22} } \\ \end{array} } \right] $$, $${\text{B}} = \left[ {\begin{array}{*{20}c} {b_{1} } \\ {b_{2} } \\ \end{array} } \right]$$,$a_{11} = 1 - \left( {M_{1} + M_{2} } \right)T_{s} \cdot K_{1} = 1 - \frac{{V_{i} + V_{o} /n}}{fL} \cdot K_{1}$,$a_{12} = - \left( {M_{1} + M_{2} } \right)T_{s} \cdot K_{2} = \frac{{V_{i} + V_{o} /n}}{fL} \cdot \frac{{K_{1} }}{{R_{s} }}$,$a_{21} = - k_{ni} \cdot \frac{{R_{so} }}{n}\left( {1 - D} \right) + k_{ni} \cdot \frac{{R_{so} }}{n}\left[ {\frac{{V_{i} + 0.5V_{o} /n}}{fL} \cdot \left( {1 - D} \right) - \frac{{V_{r} }}{{R_{so} }} \cdot \frac{n}{1 - D}} \right] \cdot K_{1}$,${a}_{22}=1-\frac{{k}_{ni}}{{R}_{s}}\cdot \frac{{R}_{so}}{n}\cdot [\frac{{V}_{i}+{0.5V}_{o} /n}{fL}\cdot \left(1-D\right)-\frac{{V}_{r}}{{R}_{so}}\cdot \frac{n}{1-D}]\cdot {K}_{1}$,${b}_{1}=-\left({M}_{1}+{M}_{2}\right){T}_{s}\cdot {K}_{3}=\frac{{V}_{i}+{V}_{o}/n}{fL}\cdot \left(1+{k}_{p}+{k}_{ni}D\right)\cdot \frac{{K}_{1}}{{R}_{s}}$,${b}_{2}={k}_{ni}-\frac{{k}_{ni}}{{R}_{s}}\cdot \frac{{R}_{so}}{n}\cdot \left[\frac{{V}_{i}+{0.5V}_{o}/n}{fL}\cdot \left(1-D\right)-\frac{{V}_{r}}{{R}_{so}}\cdot \frac{n}{1-D}\right]\cdot \left(1+{k}_{p}+{k}_{ni}D\right)\cdot {K}_{1}$, ${K}_{1}=\frac{1}{{M}_{1}{T}_{s}}\cdot \frac{1}{1+{S}_{r}-{k}_{ni}\cdot \frac{{V}_{r}}{{R}_{s}}\cdot \frac{1}{{M}_{1}{T}_{s}}}$, ${S}_{r}=\frac{L{M}_{e}}{{R}_{s}{V}_{o}/n}\cdot \frac{D}{\left(1-D\right)}={S}_{ro}\cdot \frac{D}{\left(1-D\right)}\mathrm{D}=\frac{{V}_{o}/n}{{V}_{i}+{V}_{o}/n}{k}_{p}=\frac{{R}_{2}}{{R}_{1}}$,$k_{ni} = k_{i} T_{s} = \frac{{T_{s} }}{{R_{1} C_{1} }}$, $f_{s} = \frac{1}{{T_{s} }}$, $R_{so} = \frac{{R_{o} K_{f} }}{{n_{s} }}$. ${S}_{ro}$ is the ratio of the external ramp slope to the off-time current slope multiplied by ${R}_{s}$.

## Design guidelines

### ${{\varvec{k}}}_{{\varvec{p}}}$ independent of the location of the eigenvalues

**Figure 4:**
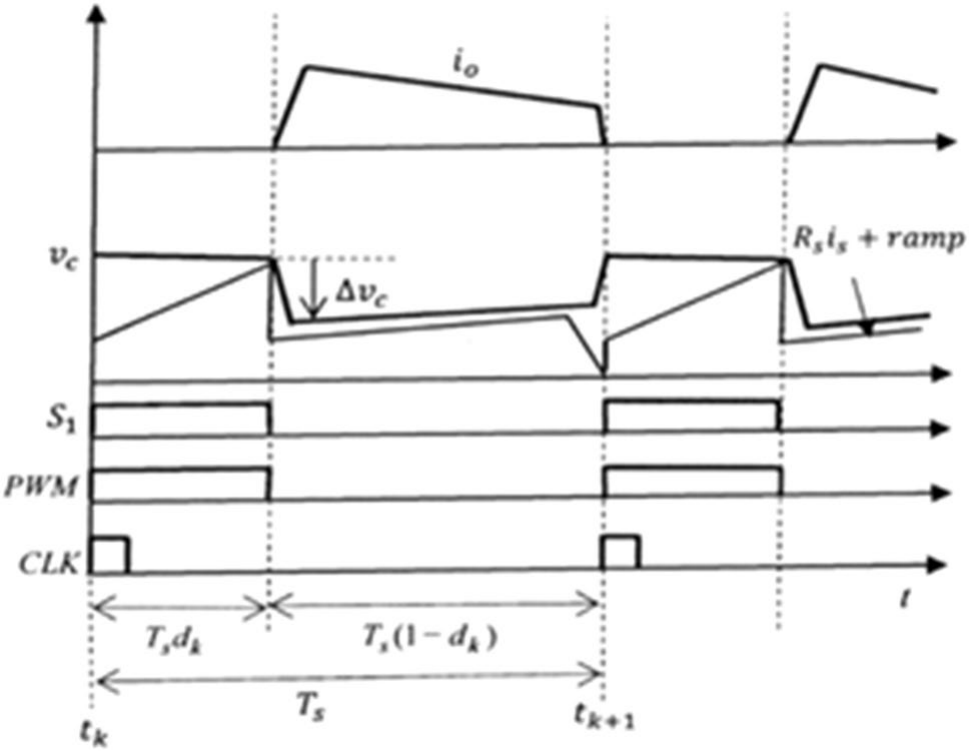
Effect of ${k}_{p}$ on the control signal ${v}_{c}$: ${\Delta v}_{c}={k}_{p}{R}_{so}{i}_{o}$.

In system Eq. (14), the terms of matrix A are independent of ${k}_{p}$. So ${k}_{p}$ does not affect the characteristics of the system. Figure 4 shows the effect of ${k}_{p}$ on the control signal ${v}_{c}$. When ${k}_{p}$ is present, it causes a change in the control signal. Proportional gain ${k}_{p}$ relates to the change of the control signal ${\Delta v}_{c}$ when the switch is off, but does not affect the slope of the control signal when the switch is on. In other words, the small signal dynamics and stability related to the slope of the control signal at the switching moment are not affected by the resistor ${R}_{2}$. Therefore, it is desirable to remove ${R}_{2}$ from the error amplifier, resulting in ${k}_{p}=0$. This is because a large ${k}_{p}$ may cause the error amplifier to malfunction.

The eigenvalues of matrix A corresponding to the poles of the system are calculated to analyze the dynamics of the entire system. The eigenvalues of A are the solutions of

15 $$ {\varvec{det}}\left( {{\mathbf{zI}} - {\mathbf{A}}} \right) = 0 $$

where I is the unit matrix.

### Selection of ${{\varvec{S}}}_{{\varvec{r}}{\varvec{o}}}$

${S}_{ro}$ Is the normalized slope of external ramp. As is well known, the current mode control is unstable in CCM when the duty cycle is greater than 0.5. A compensation ramp is added to avoid this problem. In the control strategy, the slope of the external compensation is constant. To ensure the stability of the control loop, the slope of the external ramp should be at least 50% of the downward slope of the inductor current^26^

16 $$ S_{ro} \ge 0.5 $$

**Figure 5:**
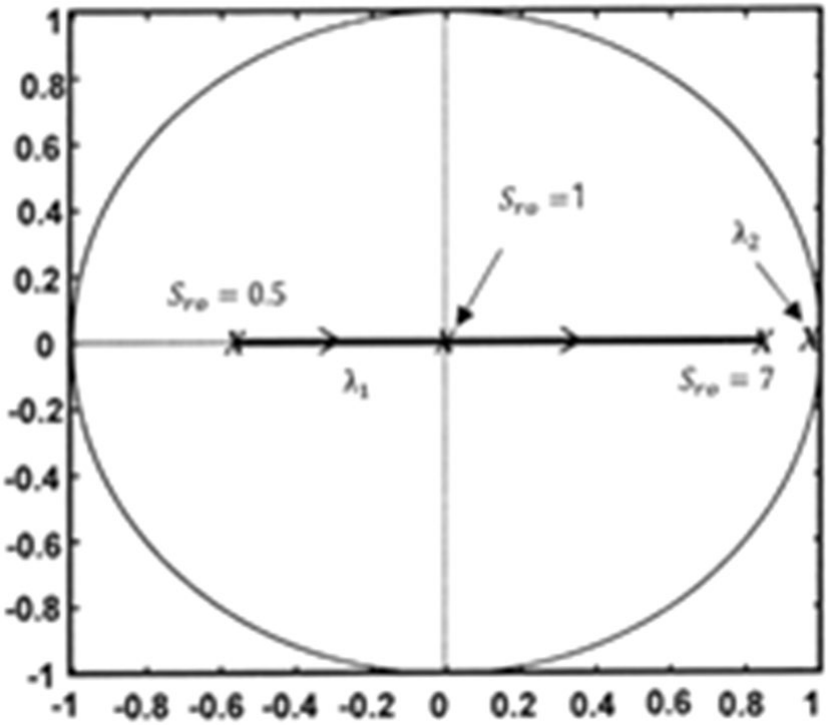
Root locus in the z-plane when varying ${S}_{ro}$ for ${k}_{p}$=0, ${k}_{ni}$=0.003, and D = 0.75.

The root locus obtained by varying the ${S}_{ro}$ value for ${k}_{p}=0$ and ${k}_{ni}=0.003$ is shown in Fig. 5. As ${S}_{ro}$ increases from 0.5 to 1, the transient response of inductor current which is dominated by ${\uplambda }_{1}$ changes from underdamping to the fastest response. This is because the eigenvalue ${\uplambda }_{1}$ shifts to the center of the unit circle. When ${S}_{ro}$ is greater than 1, the transient response of inductor current is overdamped. Selecting ${S}_{ro}$ slightly greater than 1 causes the closed-loop system to be slightly overdamped without oscillation. On the other hand, the eigenvalue ${\uplambda }_{2}$, which dominates the dynamic performance of the slower integrator state, is fixed near the unit circle. Choosing an ${S}_{ro}$ slightly greater than or equal to 1 can provide a good transient response.

### Selection of ${{\varvec{k}}}_{{\varvec{n}}{\varvec{i}}}$

**Figure 6:**
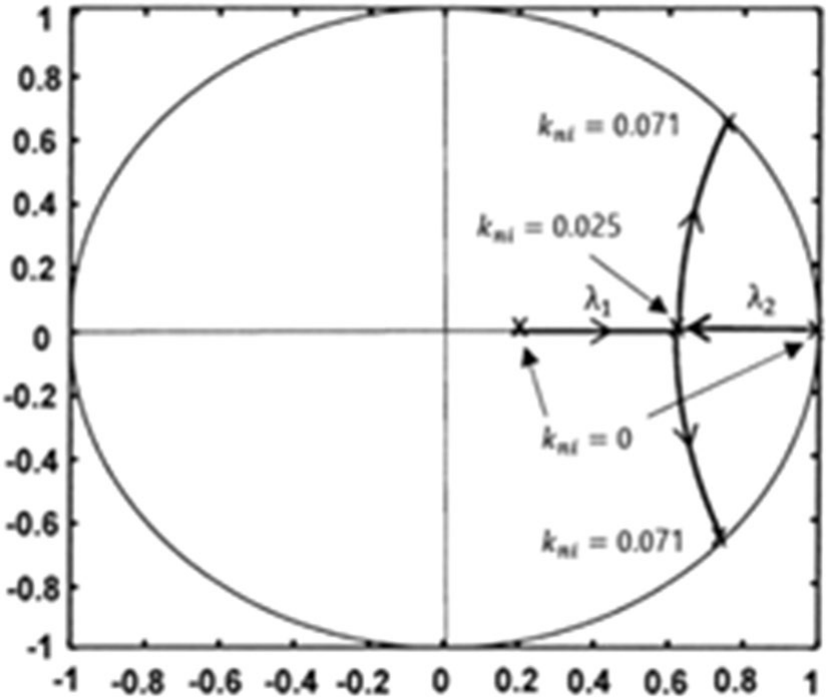
Root locus in the z-plane when varying ${k}_{ni}$ for ${k}_{p}$=0, ${S}_{ro}=1.5$ and D = 0.55.

Next, the root locus is obtained by changing ${k}_{ni}$ for ${R}_{s}=0.25\Omega$, ${R}_{so}=3\Omega , {v}_{r}=2.5 \mathrm{V}$, ${T}_{s}=10\mathrm{ \mu s}$, $\mathrm{L}=310\mathrm{ \mu H}$, ${S}_{ro}=1.5$, n = 1, ${V}_{o}=30\mathrm{ V}$, and D = 0.55. Figure 6 shows the root locus obtained by varying the integral gain ${k}_{ni}$ value for ${k}_{p}=0$. When the I gain ${k}_{ni}$ of the error amplifier changes from 0 to 0.025, the system response changes from overdamping to critical damping. The system responds faster because the slower eigenvalue ${\uplambda }_{2}$ shifts towards the center of the unit circle. When ${k}_{ni}$ is greater than 0.025, the transient response is underdamped and oscillations occur. Choosing a ${k}_{ni}$ greater than 0.071 makes the closed loop system unstable.

**Figure 7:**
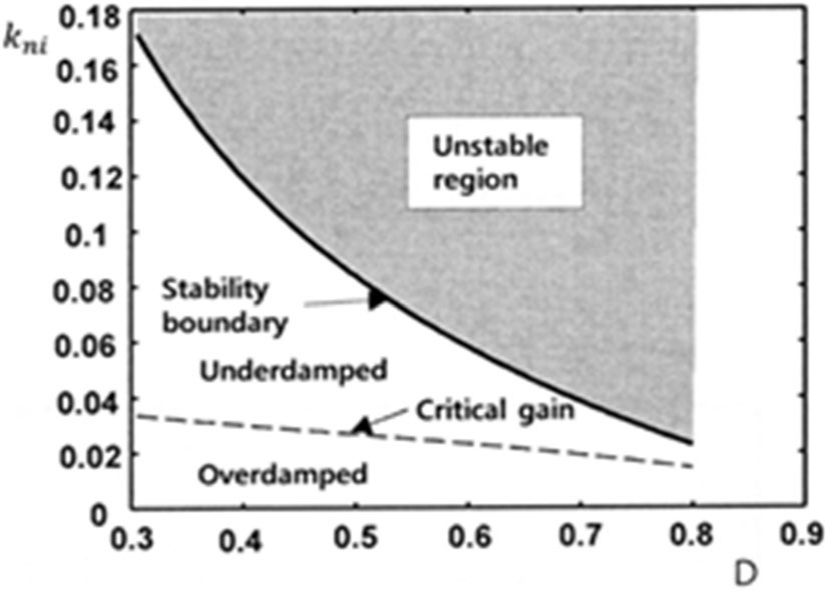
Theoretical stability boundary and critical gain curve of ${k}_{ni}$ as a function of D when ${k}_{p}$ = 0.

Figure 7 shows stability boundary and critical gain curve of ${k}_{ni}$ as a function of D. When all eigenvalues of a system are within the unit circle, the stability of the system is assured. This region is when ${k}_{ni}$ is between 0 and the stability boundary. Selecting an integral gain greater than the boundary can cause the system to be unstable. This unstable area is grayed out. It is recommended to design the integral gain so that the system response is critical damping. When the two poles coincide, i.e. ${\uplambda }_{1}$=${\uplambda }_{2}$, the system response is critically damped. We can plot the critical gain with a dashed line between the underdamped and overdamped regions by using the condition

17 $$ (a_{11} + a_{22} )^{2} - 4\left( {a_{11} a_{22} - a_{12} a_{21} } \right) = 0 $$

## Experimental evaluation

**Figure 8:**
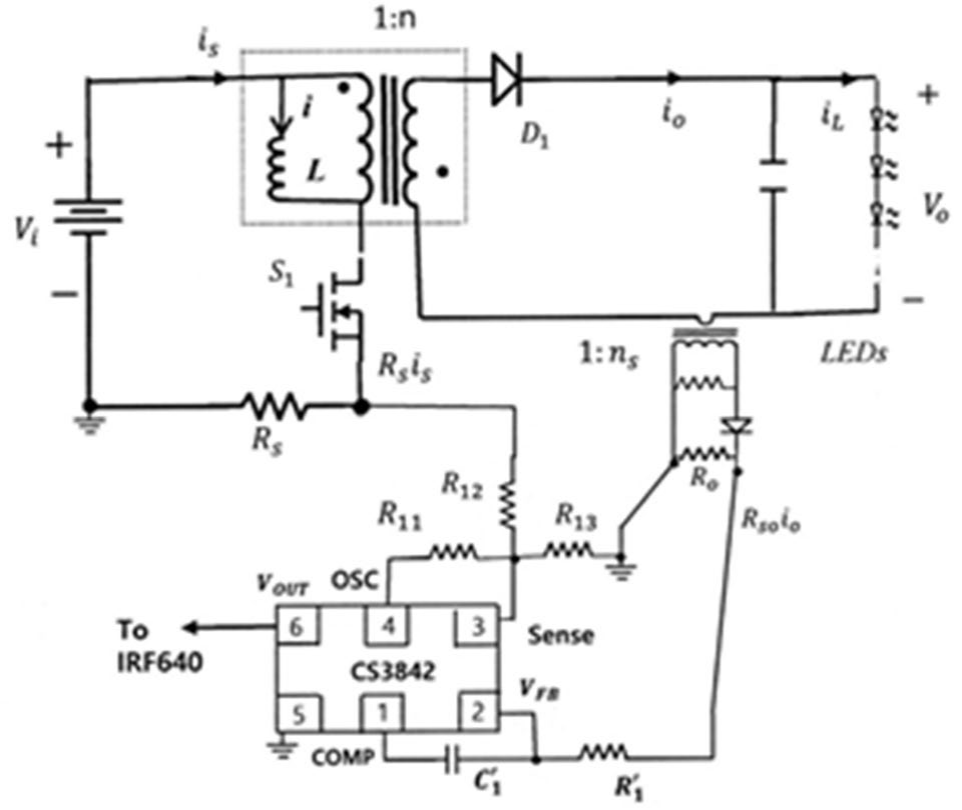
Experimental prototype circuit of CMC flyback LED driver with stabilization ramp.

For evaluation through experiments, a prototype converter circuit was implemented as shown in Fig. 8. The components and parameters of Fig. 8 are listed in Table 1. Switching is constant at 100 kHz frequency. CS3842 is used as the control IC. The output voltage is approximately 30 V (3.0 V × 10 LEDs in series). In the datasheet of CS3842, the OSC peak-to-peak swing $\mathrm{\Delta V}$ is 1.8 V^27^. The rising slope of OSC is ${M}_{e}=\Delta V/{T}_{s}=1.8\times {10}^{5}$. For the operating point and system parameters, ${S}_{r0}$ is $\frac{L{M}_{e}}{{R}_{s}{V}_{o}}\times \frac{{R}_{12}}{{R}_{11}}$=$\frac{310X{10}^{-6}\times 1.8X{10}^{5}}{0.25\times 30}\times \frac{{R}_{12}}{{R}_{11}}=7.44\times \frac{{R}_{12}}{{R}_{11}}$. To design ${S}_{r0}$ to be 1.5, slightly greater than 1, the values of ${R}_{11}$ and ${R}_{12}$ are chosen to be 50 kΩ and 10 kΩ, respectively. In the datasheet^27^, the Sense signal should be limited to within 1 V. The maximum value of OSC voltage is 2.8 V. If the maximum value of the primary-side inductor current is set to 3.5A, the maximum value of the Sense signal is $\mathrm{max}\left(\mathrm{OSCX}\frac{1}{5}+{R}_{s}{i}_{s}\right)XSF=\left(2.8\mathrm{X}\frac{1}{5}+0.25X3.5\right)XSF=1 V.$ So, SF is 0.7. In Fig. 8, the Sense signal can be derived as $\mathrm{OSC}\cdot \frac{{R}_{12}{R}_{13}}{{R}_{12}+{R}_{13}}/({R}_{11}+\frac{{R}_{12}{R}_{13}}{{R}_{12}+{R}_{13}})+{R}_{s}{i}_{s}\cdot \frac{{R}_{11}{R}_{13}}{{R}_{11}+{R}_{13}}/({R}_{12}+\frac{{R}_{11}{R}_{13}}{{R}_{11}+{R}_{13}})$. Therefore, $\frac{{R}_{11}{R}_{13}}{{R}_{11}+{R}_{13}}/({R}_{12}+\frac{{R}_{11}{R}_{13}}{{R}_{11}+{R}_{13}})=\mathrm{SF}\approx$0.7. Substituting ${R}_{11}=50 \mathrm{k\Omega }$ and ${R}_{12}=10\mathrm{ k\Omega }$ into this equation gives ${R}_{13}=50 \mathrm{k\Omega }$.

**Table 1:** Components and system parameters used in the experiment.

${f}_{s}$	100 kHz	${C}_{1}^{\mathrm{^{\prime}}}$	47 nF
D	0.55	${S}_{1}$	IRF640
L	310 $\mathrm{\mu H}$	${D}_{1}$	MBRF10100CT
${R}_{s}$	0.25 Ω	LEDs	SZ5-W0-M0-00
${V}_{o}$	30 V	Load current	833 mA
${R}_{o}$	150 Ω	${R}_{11}$	50 kΩ
n	1	${R}_{12}$	10 kΩ
${n}_{s}$	50	${R}_{13}$	50 kΩ
${K}_{f}$	1	${V}_{i}$	25 V
${S}_{ro}$	1.5	Control IC	CS3842

When the integral gain ${k}_{ni}$ is multiplied by the scale factor (SF), the characteristics of the real system become the same as the theoretical analysis^20^. So, the actual integral gain ${k}_{ni}^{^{\prime}}$ in the output feedback loop becomes ${k}_{ni}XSF$. In the experimental circuit, the output signal of the error amplifier is reduced by 1/3 by the voltage divider resistors. So, the designed integral gain in the output feedback loop ${k}_{ni}^{^{\prime}}$ is $\frac{1}{3}\times \frac{{T}_{s}}{{R}_{1}^{^{\prime}}{C}_{1}^{^{\prime}}}$. Setting ${C}_{1}^{^{\prime}}=47\mathrm{ nF}$ gives ${k}_{ni}=\frac{{k}_{ni}^{^{\prime}}}{SF}=\frac{1}{0.714X3}\times \frac{{10}^{-5}}{{R}_{1}^{^{\prime}}X47X{10}^{-9}}$ =$\frac{0.0993X{10}^{3}}{{R}_{1}^{^{\prime}}}$.

**Figure 9:**
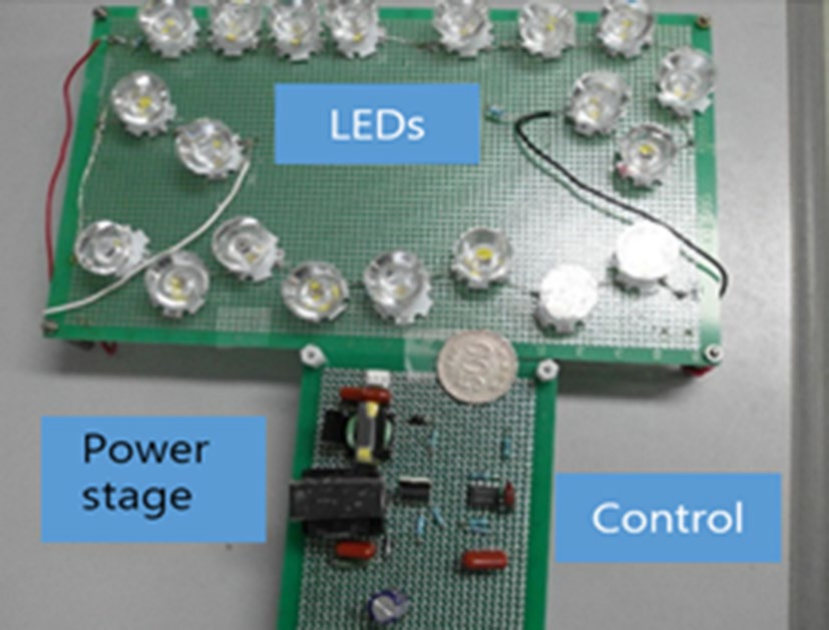
Photo of the experimental implementation.

**Figure 10:**
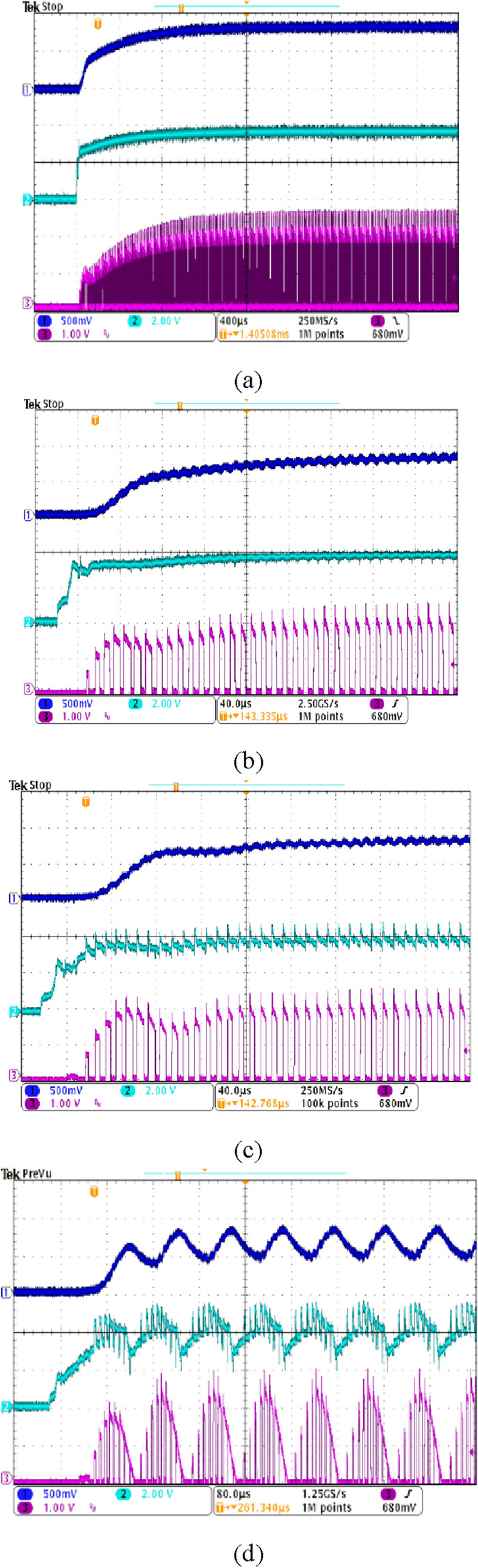
Start-up responses when the integral gain ${k}_{ni}$ changes, (**a**) ${k}_{ni}\approx 0.0083\left( {R}_{1}^{^{\prime}}=12 k\right),$ (**b**) ${k}_{ni}\approx 0.027({R}_{1}^{^{\prime}}=3.7 k)$, (**c**) ${k}_{ni}\approx 0.055\left( {R}_{1}^{^{\prime}}=1.8 k\right),$ and (**d**) ${k}_{ni}\approx 0.1\left( {R}_{1}^{^{\prime}}=1 k\right).$ Vertical scale: top traces-load current ${i}_{L}$(0.5 A/div.); middle traces-error amplifier output voltage COMP (2 V/div); bottom traces- transformer secondary output current ${i}_{o}$(1 A/div).

**Figure 11:**
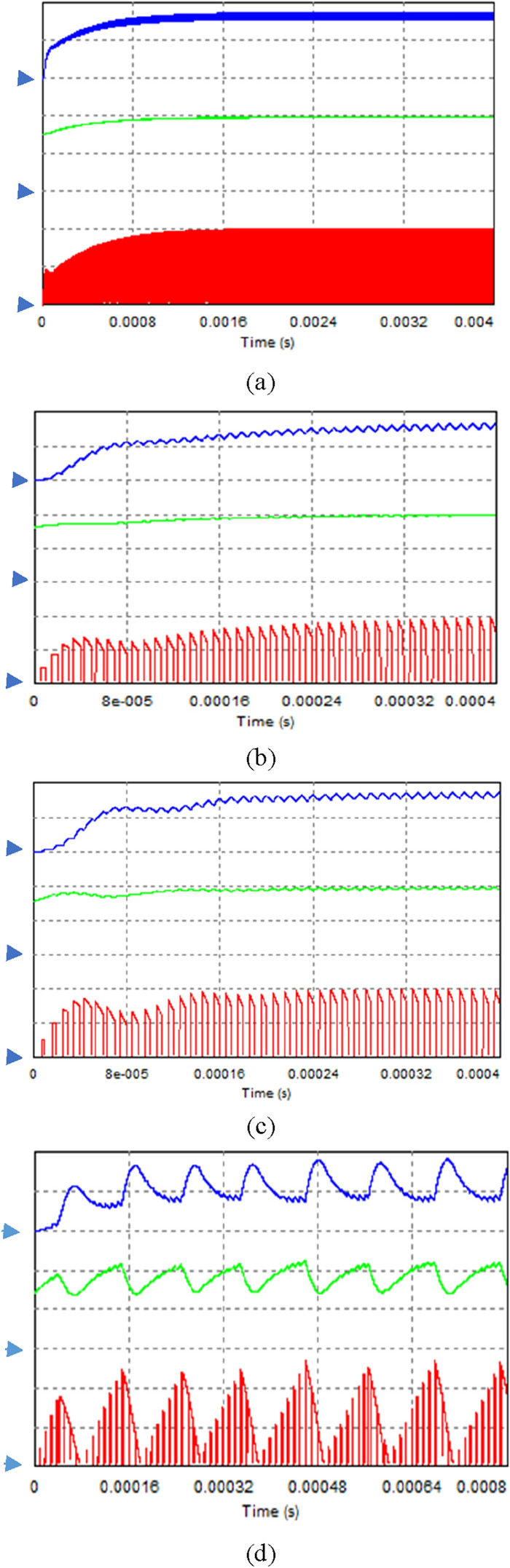
Simulated waveforms at start-up, (**a**) ${k}_{ni}\approx 0.0083\left( {R}_{1}^{^{\prime}}=12 k\right),$ (**b**) ${k}_{ni}\approx 0.027({R}_{1}^{^{\prime}}=3.7 k)$, (**c**) ${k}_{ni}\approx 0.055\left( {R}_{1}^{^{\prime}}=1.8 k\right),$ and (**d**) ${k}_{ni}\approx 0.1\left( {R}_{1}^{^{\prime}}=1 k\right).$ Vertical scale: top traces-load current ${i}_{L}$(0.5 A/div.); middle traces-error amplifier output voltage COMP (2 V/div); bottom traces- transformer secondary output current ${i}_{o}$(1 A/div).

**Figure 12:**
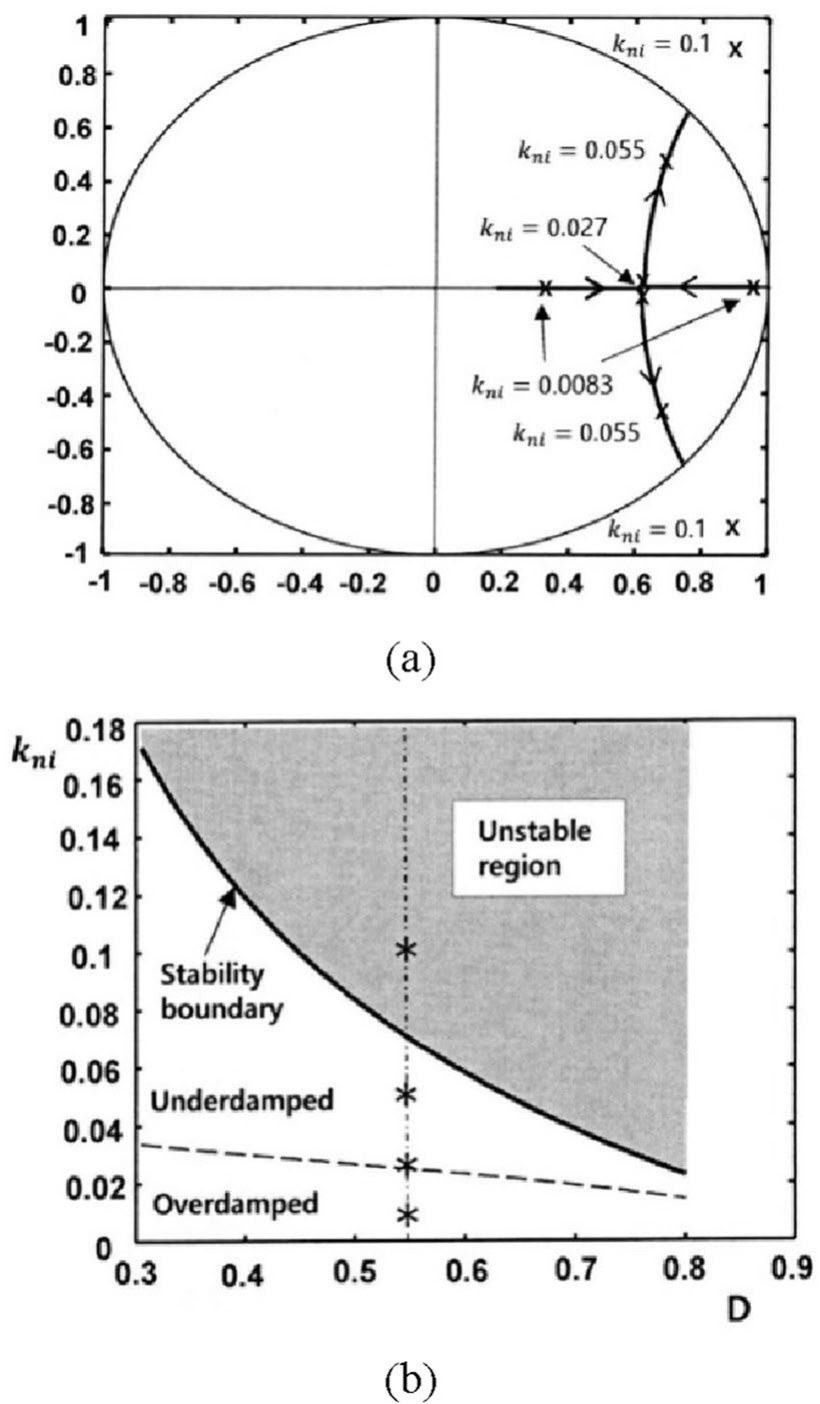
Experimental points: (**a**) pole locations in the z-plane and (**b**) representations in the ${k}_{ni}$ versus D plane.

In the experiment, 10 LEDs were connected in series to provide a load voltage of about 30 V. Figure 9 shows a picture of the implementation hardware. As shown in Fig. 10, the LED current ${i}_{L}$, the output of the error amplifier COMP, and the transformer secondary current ${i}_{o}$ are measured for the starting transient when the integral gain is changed. The overall response time is faster as the integral gain varies from 0.0083 to 0.027. An optimal transient response is measured when ${k}_{ni}$ is 0.027, that is, near the point ${\lambda }_{1}$ = ${\lambda }_{2}$. By changing the integral gain to 0.055, the transient response of the system exhibits slight oscillations. Increasing ${k}_{ni}$ to 0.1 results in an unstable system with pole locations outside the unit circle. At ${k}_{ni}=0.1$, the pole positions in the z-plane are $0.9\pm \mathrm{j}0.87$. The z-plane pole positions are expressed as the s-plane pole positions as follows^28^: $\mathrm{z}={e}^{s{T}_{s}}{|}_{s=\sigma \pm jw}={e}^{\sigma {T}_{s}}/\underset{\_}{\pm w{T}_{s}}$ =$1.25/\underset{\_}{\pm 0.768}$. Since $\mathrm{w}{T}_{s}$=0.768, the closed-loop oscillation frequency is ${f}_{osc}=\frac{0.768}{2\pi {T}_{s}}=0.122{f}_{s}$. So, the oscillation period becomes $\frac{1}{{f}_{osc}}=8.2{T}_{s} \approx 82 \mathrm{\mu s}$, which is consistent with the measured unstable waveform as shown in Fig. 10d. The observed experimental results show excellent agreement with the root locus analysis. Figure 11 shows the simulated waveforms using the PSIM simulation program when the integral gain of the error amplifier is changed. The soft start time simulation of the COMP signal is omitted. The experimental points of the two analysis planes are shown in Fig. 12. Comparison between the proposed design and the previous design^5^ is shown in Table 2. The typical efficiency of this flyback LED driver with an output power of 25 W is over 90%.

**Table 2:** Comparison between proposed and previous current-mode controller designs.

Proposed design	Previous design ref. ^5^
Discrete time domain design	Continuous time domain design
Simultaneous error amplifier and current-loop designs	Error amplifier design after designing current loop
Constant slope compensation	Variable slope compensation
Internal ramp compensation signal OSC of CS3842 used for the current loop	External exponential ramp compensation signal used for the current loop
Simple hardware implementation: 3 resistors for the current loop, and 1 resistor and 1 capacitor for the error amplifier	Complex hardware implementation: 2 transistors, 2 diodes, 7 resistors and 3 capacitors for the current loop, and 1 opamp, 6 resistors and 3 capacitors for the error amplifier

## Conclusion

A sequential process of modeling for a CMC flyback LED driver with a stabilizing ramp is proposed. Discrete time equations representing the behavior of the CMC flyback converter are derived. Then these equations are linearized with respect to the steady-state operating point. At the moment of switching, the control law is also linearized with respect to the operating point. In the next step, a linearized CMC flyback LED driver with a stabilizing ramp is derived by combining the two models of a linearized flyback converter and a linearized switching control law.

The inner feedback loop uses a current mode controller with ramp compensation slope. A PI compensator is used as an error amplifier in the output feedback loop. Here is a suggested step-by-step procedure for selecting feedback loop gains such as slope ratio and integral gain in the output feedback loop. First, ${S}_{r0}$ is chosen to be 1 or a slightly greater value. Second, ${k}_{p}$ is set to zero. The integral gain ${k}_{ni}$ is designed to be close to the critical gain obtained using (17). Third, the maximum value of the comparator input voltage requires a limit^27^. To accommodate this, SF is calculated. This SF is less than 1. The actual integral gain ${k}_{ni}^{^{\prime}}$ is calculated by multiplying ${k}_{ni}$ by SF.

Using the step-by-step procedure described above, the design engineer can easily select the appropriate feedback gains for a well-characterized system. The proposed control can be implemented with a very simple compensation circuit. The validity of the proposed design is confirmed through the presented experimental results.

## Data Availability

All data generated or analysed during this study are included in this published article.

## References

[1] Molavi N, Farzanehfard H (2022). Load-independent hybrid resonant converter for automotive LED driver applications. IEEE Trans. Power Electron..

[2] Mukherjee S, Yousefzadeh V, Sepahvand A, Doshi M, Maksimovic D (2021). A two-stage automotive LED driver with multiple outputs. IEEE Trans. Power Electron..

[3] Soares GM, Alonso JM, Braga HAC (2018). Investigation of the active ripple compensation technique to reduce bulk capacitance in offline flyback-based LED drivers. IEEE Trans. Power Electron..

[4] He Q, Luo Q, Wei Y, Sun P (2021). A variable inductor controlled single-stage AC/DC converter for modular multi-channel LED driver. IEEE Trans. Energy Convers..

[5] Lamar DG, Arias M, Rodriguez A, Fernandez A, Hernando MM, Sebastian J (2013). Design-oriented analysis and performance evaluation of a low-cost high-brightness LED driver based on flyback power factor corrector. IEEE Trans. Ind. Electron..

[6] Li H, Li S, Xiao W (2021). Single-phase LED driver with reduced power processing and power decoupling. IEEE Trans. Power Electron..

[7] Abdelmessih GZ, Alonso JM, Spode ND, Dalla Costa MA (2022). High-efficient electrolytic-capacitor-less offline LED driver with reduced power processing. IEEE Trans. Power Electron..

[8] Tian H, Chen M, Liang G, Xiao X (2022). Single-phase rectifier with reduced common-mode current, auto-PFC, and power decoupling ability. IEEE Trans. Power Electron..

[9] Cheng HL, Chang YN, Chang CH, Hsieh SY, Cheng CA (2019). A novel high-power-factor AC/DC LED driver with dual flyback converters. IEEE J. Emerg. Sel. Top. Power Electron..

[10] Kim MG (2021). A study on optimal selection of inductance for power factor improvement of buck AC/DC LED Driver with wide input voltage range. Trans. Korean Inst. Power Electron..

[11] Lee SW, Do HL (2017). A single-switch AC-DC LED driver based on a boost-flyback PFC converter with lossless snubber. IEEE Trans. Power Electron..

[12] Zhang X, Cai H, Guan Y, Han S, Wang Y, Dalla Costa MA, Alonso JM, Xu D (2021). A soft-switching transformer-less step-down converter based on resonant current balance module. IEEE Trans. Power Electron..

[13] Liu PJ, Hsu YC, Hsu SR (2018). Drain-voltage balance and phase-shifted PWM control schemes for high-efficiency parallel-string dimmable LED drivers. IEEE Trans. Ind. Electron..

[14] Liu X, Wan Y, Dong Z, He M, Zhou Q, Tse CK (2020). Buck-boost-buck-type single-switch multistring resonant LED driver with high power factor and passive current balancing. IEEE Trans. Power Electron..

[15] Middlebrook RD (1989). Modeling current-programmed buck and boost regulators. IEEE Trans. Power Electron..

[16] Lee FC, Iwens RP, Yu Y, Triner JE (1979). Generalized computer-aided discrete-time modeling and analysis of dc-dc converters. IEEE Trans. Industr. Electron. Contr. Instrum..

[17] Jung YS, Kim MG (2012). Time-delay effects on DC characteristics of peak current controlled power LED driver. J. Power Electron..

[18] Feng W, Lee FC, Mattavelli P (2014). Optimal trajectory control of LLC resonant converter for LED PWM dimming. IEEE Trans. Power Electron..

[19] Kim MG (2015). Proportional-Integral (PI) compensator design of duty-cycle-controlled buck LED driver. IEEE Trans. Power Electron..

[20] Kim MG (2018). High-performance current-mode-controller design of buck LED driver with slope compensation. IEEE Trans. Power Electron..

[21] Menke MF, Seidel AR, Tambara RV (2019). LLC LED driver small-signal modeling and digital control design for active ripple compensation. IEEE Trans. Ind. Electron..

[22] Verghese GC, Elbuluk ME, Kassakian JG (1986). A general approach to sampled-data modeling for power electronic circuit. IEEE Trans. Power Electron..

[23] Kim MG, Youn MJ (1991). A discrete time domain modeling and analysis of controlled series resonant converter. IEEE Trans. Ind. Electron..

[24] Kim MG, Youn MJ (1991). An energy feedback control of series resonant converter. IEEE Trans. Power Electron..

[25] Kim MG, Lee DS, Youn MJ (1991). A new state feedback control of resonant converters. IEEE Trans. Ind. Electron..

[26] Huber L, Gang L, Jovanovic MM (2010). Design-oriented analysis and performance evaluation of buck PFC front end. IEEE Trans. Power Electron..

[27] Cherry Semiconductor Corp, CS3842B datasheet. http://www.onsemi.com/pub/Collateral/CS3842B-D.PDF (2001).

[28] Phillips CL, Nagle HT (1984). Digital Control System Analysis and Design.

